# Post-COVID-19 Encephalitis With Claustrum Sign Responsive to Immunomodulation

**DOI:** 10.7759/cureus.35363

**Published:** 2023-02-23

**Authors:** Mian Bilal Humayun, Sarah Khalid, Hamza Khalid, Warda Zahoor, Waseem T Malik

**Affiliations:** 1 Internal Medicine, Shifa International Hospital, Islamabad, PAK; 2 Neurology, Shifa International Hospital, Islamabad, PAK

**Keywords:** coronavirus disease 2019, covid-19, intravenous immunoglobulin, intravenous immunoglobulins (ivig), pediatric, autoimmune, neurology, encephalitis

## Abstract

We describe a case of encephalitis following coronavirus disease 2019 (COVID-19) in a six-and-a-half-year-old girl who presented with acute onset confusion and jerky movements of the limbs. The patient was unvaccinated for COVID-19. Subsequent magnetic resonance imaging revealed a bilateral "claustrum sign" on T2 and fluid-attenuated inversion recovery (FLAIR) images and electroencephalogram reported moderate diffuse encephalopathy. The patient tested negative for COVID-19 by polymerase chain reaction, had positive serology for COVID-19 indicating past infection, and had a negative autoimmune panel and infectious workup. She was treated on the lines of post-infectious encephalitis with immunomodulatory therapies such as high-dose intravenous steroids and intravenous immunoglobulins. She responded significantly and had complete resolution of her symptoms; therefore, further supporting the suspicion of an immune-mediated etiology. Cases of post-COVID-19 encephalitis have been reported all over the world; however, most cases are based on speculation and temporal associations and therefore more research is required to optimize treatment guidelines.

## Introduction

The objective of this clinical case report is to facilitate the identification and appropriate management of post-coronavirus disease 2019 (COVID-19) encephalitis. In a post-COVID-19 world, the incidence of COVID-19-associated syndromes is likely to increase; hence, clinicians must keep this branch of diseases under consideration in future clinical practice. Our case is of a young girl who had an asymptomatic COVID-19 infection and now presented with neurological symptoms after two months. Specific findings on MRI were suggestive of post-COVID-19 encephalitis. The patient was managed under this diagnosis with immunomodulatory therapy, which resulted in the improvement of symptoms.

## Case presentation

Methods

Informed consent was obtained to report this case. MRI of the brain was performed using axial T1, T2, fluid-attenuated inversion recovery (FLAIR), diffusion-weighted imaging (DWI), and T1 post-contrast sequences. Informed consent was obtained from both parents regarding the publishing of this case. Approval was obtained from the internal review board of Shifa International Hospital prior to submission.

Case

We describe a case of a six-and-a-half-year-old girl with no previously known comorbidity and unvaccinated for COVID-19, who presented with temporal headache, strange behavior such as excessive talking and laughing, as well as auditory and visual hallucinations. She also complained of double vision but there was no photophobia, fever, or neck stiffness. The patient had myoclonic jerks in her right leg, which gradually progressed to involve the entire body but she remained conscious throughout these episodes. History revealed a member of the household tested positive for COVID-19 via polymerase chain reaction (PCR) two months ago; however, the patient herself remained asymptomatic and was not tested. She had no history of febrile seizures or epilepsy.

On examination, the Glasgow Coma Scale score was 13/15, pupils were 2 mm round and equally reactive to light, extraocular movements were intact, no gross facial asymmetry was noted, the tongue was central, and the plantar reflexes were down-going equally. She was found to have nystagmus and finger nose ataxia, she was unable to walk without support, and had a broad-based ataxic gait.

During her hospital stay, she had multiple episodes of generalized tonic-clonic seizures with loss of consciousness, uprolling of eyes, and hypoxia with oxygen saturation (SpO2) lowest at 60%, hence requiring oxygen therapy. Post-seizure, her cranial reflexes, power, and tone were normal. Finger nose ataxia and a broad-based ataxic persisted and she also had poor oral intake due to slow chewing.

Investigations

Upon admission, a work-up was initiated to identify a possible etiology of seizures. A complete blood count was done, which showed thrombocytosis (platelet count: 787,000) and mild microcytic anemia (hemoglobin: 10.8; mean corpuscular volume: 72.9). Serum electrolytes and glucose levels were done to exclude any obvious metabolic causes of seizures. A urine toxicology profile was also done, which showed no signs of any substance use. Markers of acute inflammation such as white cell count with differential (WBC: 8970/UL), C-reactive protein (CRP: 0.60mg/L), erythrocyte sedimentation rate (ESR: 16 mm), and serum ferritin (140 ng/ml) were within normal limits; this, along with the absence of clinical signs, including fever, made acute bacterial causes such as meningoencephalitis less likely; however, aseptic causes needed to be further investigated [1]. Cerebrospinal fluid (CSF) analysis was done, which showed <05 white cells along with appropriate levels of glucose and protein (Table 1). CSF culture along with herpes simplex virus (HSV) type 1 and 2 PCRs were negative.

**Table 1:** CSF routine examination

CSF routine examination	Patient value	Reference range
Color	Colorless	Colorless
Appearance	Crystal clear	Crystal clear
Coagulum	Not present	Not present
Xanthochromia	Not present	Not present
White blood cells	<5 cell/uL	<5 cells/uL
Red blood cells	Nil	Nil
Glucose	73.9	50-80
Protein	15.8	15-40

Liver function tests as well as serum ceruloplasmin were also done with hepatic encephalopathy and Wilson’s disease in mind, and both were within normal limits. Autoimmune etiology was investigated, including systemic lupus erythematosus, rheumatic fever, and autoimmune receptor encephalitis, all of which showed no abnormality (Tables 2, 3). CSF reverse transcription-PCR (RT-PCR) was not performed at the time due to the unavailability of the test.

**Table 2:** Autoimmune encephalitis antibodies NMDA: N-methyl-D-aspartate; GABAb: gamma-aminobutyric acid B.

Autoimmune receptor antibodies	Patient value
NMDA receptor	Negative
CASPR2	Negative
Glutamate receptor	Negative
Leucine-rich glioma-inactivated protein 1 antibodies	Negative
Dipeptidyl-aminopeptidase-like protein 6	Negative
GABAb receptor	Negative

**Table 3:** Systemic lupus erythematosus workup SLE: systemic lupus erythematosus; dsDNA: double-stranded DNA.

SLE antibody	Patient value	Reference range
Nucleosome	Negative	Negative
Histone	Negative	Negative
dsDNA	Negative	Negative
C3	1.61 G/L	1-14 years; female: 0.82-1.73 G/L
C4	0.35 G/L	1-14 years; female: 0.13-0.46 G/L

**Figure 1:**
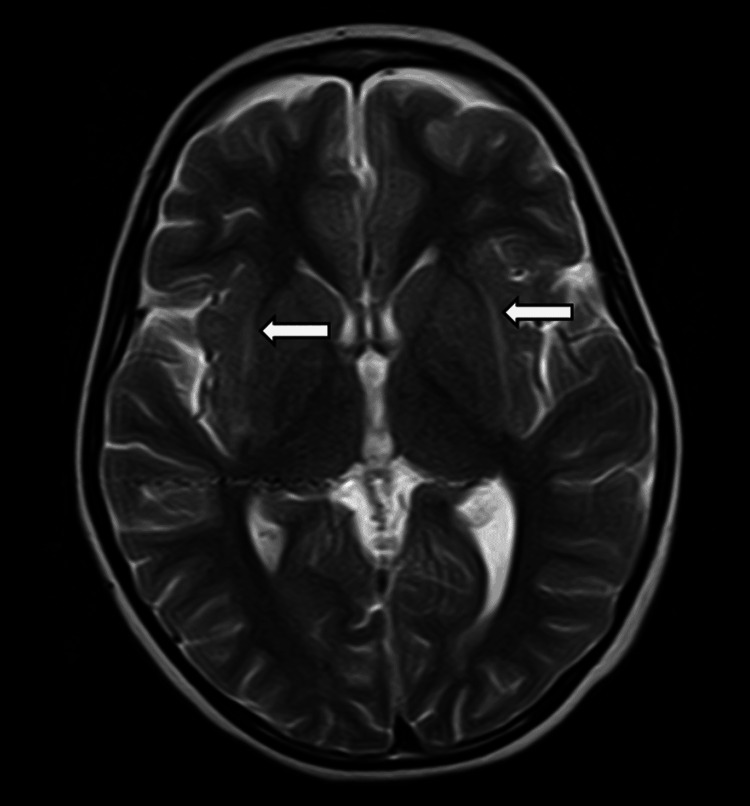
Magnetic resonance imaging of the brain (T2-weighted) White arrows pointing to the hyperintense claustrum.

**Figure 2:**
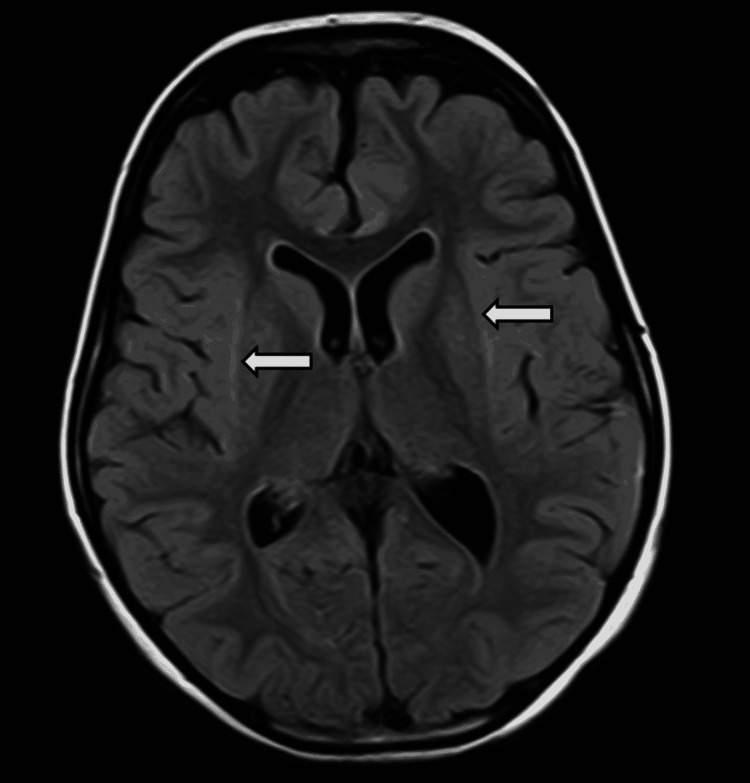
Magnetic resonance imaging of the brain (fluid-attenuated inversion recovery) White arrows pointing to the hyperintense claustrum.

MRI was done on admission, which ruled out any space-occupying lesions and showed no signs of leptomeningeal enhancement or cerebrovascular disease. It showed symmetrical T2 and FLAIR hyperintense signals showing diffusion restriction in bilateral external capsules (Figures 1, 2). The described "claustrum sign" raised the suspicion of para infectious encephalitis [2].

COVID-19 RT-PCR taken by nasopharyngeal swab on admission was negative; however, anti-SARS-CoV-2 antibodies were markedly raised, which supported her history of having an asymptomatic infection two months ago. Electroencephalogram that had been carried out showed background activity consisting of moderate amplitude 4 Hz theta activity with intermixed delta activity, signifying moderate diffuse encephalopathy. Therefore, a diagnosis of post-COVID-19 autoimmune encephalitis was made, as the patient had a subacute onset of her symptoms with new focal CNS findings, seizures that were not explained by a previously known seizure disorder, and the exclusion of alternative causes [3].

Management

The patient was given intravenous levetiracetam 12.5 mg/kg/dose twice daily, syrup clonazepam 0.025 mg/kg/day, oral phenytoin 4.5 mg/kg/day, and intravenous Dormicum 0.1 mg/kg as per need. The patient continued to have seizures, so levetiracetam was increased to 20 mg/kg/dose; however, the generalized tonic-clonic seizures remained uncontrolled. She was not given any antibiotics or antivirals as the infective markers were in a normal range. The patient was then given intravenous methylprednisolone and intravenous immunoglobulin (IVIG) (0.4 mg/kg for two doses), which achieved adequate seizure control and improvement of her symptoms. Post immunomodulation, she remained seizure-free. Upon discharge, she was able to walk with support and was sent home with an emphasis on physiotherapy and rehabilitation. She presented in the outpatient department with marked improvement in her symptoms. A follow-up EEG after five months showed a relatively normal background but no well-formed posterior dominant rhythm was demonstrated. There were some subtle changes in the runs of the delta, which were sustained but overall there was a significant improvement compared to the previous EEG. Clinically, there was a complete resolution of her symptoms with a normal gait.

## Discussion

Our case describes a young girl with post-infectious encephalitis secondary to the COVID-19 infection, as neurological complications, which are postulated to be immune-mediated following COVID-19, are becoming increasingly frequent [4-7].

The possibility of infectious encephalitis had been ruled out through extensive testing, which included metabolic, septic, autoimmune, and drug abuse profiles. Furthermore, the bilateral "claustrum" sign that appeared on MRI imaging could be a sign of post-infectious autoimmune encephalitis, as other diseases, such as Wilson’s disease, which present similarly, had been ruled out [8,9]. The etiology of post-COVID-19 encephalitis is only theorized as of now; however, in our case, the exclusion of an active COVID-19 infection, a positive serology, and responsiveness to steroids and IVIG signify an autoimmune etiology, similar to other cases that have been reported [7,10]. However, additional research is required for optimal treatment guidelines.

Although the exact mechanism of this disease is unknown, we are under the assumption that it is associated with the formation of auto-antibodies due to the presence of the claustrum sign, which is seen in autoimmune encephalitis. If we assume that our patient had an asymptomatic infection around the same time as her house member i.e., two months ago, we are more inclined to believe this is an auto-immune condition as the timing between infection and presentation of symptoms is similar to other known parainfectious etiologies in children such as rheumatic fever and post-infectious acute cerebellar ataxia [11,12]. Due to the presumed autoimmune nature of the disease, the safety of COVID-19 vaccination in such cases also requires investigation due to the potential risk of increasing cross-reacting antibodies.

## Conclusions

Cases of post-infectious encephalitis following COVID-19 have been reported around the world and presumed to be autoimmune; however, the exact mechanism is yet unknown. Claustrum sign on MRI has been seen in multiple cases reported; however, the sensitivity of this sign requires further investigation. There has been a marked response to immunomodulation, thus aiding in the suspicion of an autoimmune etiology of this disease; however, more research is required to optimize treatment guidelines.
